# From student to steward: the Interdisciplinary Program in Neuroscience at Georgetown University as a case study in professional development during doctoral training

**DOI:** 10.3402/meo.v19.22623

**Published:** 2014-07-07

**Authors:** Lauren Ullrich, Sonya B. Dumanis, Tanya M. Evans, Alexis M. Jeannotte, Carrie Leonard, Summer J. Rozzi, Caitlin M. Taylor, Karen Gale, Jagmeet S. Kanwal, Kathleen A. Maguire-Zeiss, Barry B. Wolfe, Patrick A. Forcelli

**Affiliations:** 1 Interdisciplinary Program in Neuroscience, Georgetown University, Washington, DC, USA; 2 Department of Neurology, Georgetown University, Washington, DC, USA; 3 Department of Neuroscience, Georgetown University, Washington, DC, USA; 4 Department of Pediatrics, Georgetown University, Washington, DC, USA; 5 Department of Biochemistry and Molecular & Cellular Biology, Georgetown University, Washington, DC, USA; 6 Department of Biology, Georgetown University, Washington, DC, USA; 7 Department of Pharmacology & Physiology, Georgetown University, Washington, DC, USA

**Keywords:** neuroscience, professional development, training, communication, teaching, public outreach, leadership, PhD, graduate school

## Abstract

A key facet of professional development is the formation of professional identity. At its most basic level, professional identity for a scientist centers on mastery of a discipline and the development of research skills during doctoral training. To develop a broader understanding of professional identity in the context of doctoral training, the Carnegie Initiative on the Doctorate (CID) ran a multi-institutional study from 2001 to 2005. A key outcome of the CID was the development of the concept of ‘stewards of the discipline’. The Interdisciplinary Program in Neuroscience (IPN) at Georgetown University participated in CID from 2003 to 2005. Here, we describe the IPN and highlight the programmatic developments resulting from participation in the CID. In particular, we emphasize programmatic activities that are designed to promote professional skills in parallel with scientific development. We describe activities in the domains of leadership, communication, teaching, public outreach, ethics, collaboration, and mentorship. Finally, we provide data that demonstrate that traditional metrics of academic success are not adversely affected by the inclusion of professional development activities in the curricula. By incorporating these seven ‘professional development’ activities into the required coursework and dissertation research experience, the IPN motivates students to become stewards of the discipline.

A key facet of early professional development is the formation of a professional identity. At its core, professional identity for a scientist during graduate training centers on: 1) scholarship built from understanding of a discipline, 2) mastery of the literature, and 3) the acquisition of research skills. However, regardless of ultimate career path, there are a variety of additional skills and best practices that, when instilled early during the training period, may be beneficial to the practitioner and also serve to both preserve and grow the research field.

From 2001 through 2005, the Carnegie Initiative on the Doctorate (CID) implemented a study to examine a broader understanding of professional identity. The goal was to focus on doctoral training as an instrument for producing ‘stewards of the discipline’ (1). Facets of stewardship that were identified in the CID study include taking responsibility for the future of one’s chosen discipline by generating new knowledge, integrating the new knowledge into the existing framework of one’s discipline, and conserving and transforming the knowledge by disseminating it among colleagues and students, as well as sharing its essence with lay people (Fig. 1). Thus, although doctoral training is strongly focused on mastery of a discipline and the development of research skills, becoming a ‘steward of the discipline’ requires more than accumulation of knowledge.

**Fig. 1:**
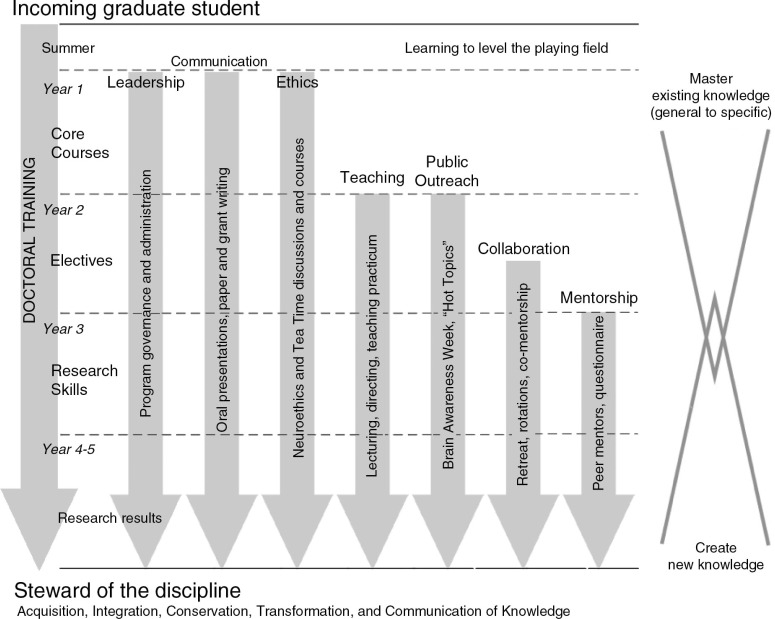
Schematic representation of activities that IPN graduate students participate in from entry into the program through the end of doctoral training, emphasizing the different facets of stewardship that we attempt to foster during the period of doctoral training.

The Interdisciplinary Program in Neuroscience (IPN) at Georgetown University (GU) was one of 15 neuroscience doctoral programs chosen for participation in the CID. The IPN aims to produce neuroscientists with a comprehensive and well-rounded education in an inherently multi-disciplinary field, which encompasses cellular, molecular, systems, and cognitive approaches. Although dissertation research constitutes the core of doctoral training, participation in the CID became the vehicle for initiating a series of discussions and programmatic changes that have led the program to include the broader development of professional identity as a core component of graduate education.

Although the majority of doctoral trainees in neuroscience and other biomedical sciences conduct post-doctoral research after the completion of the degree (2, 3), only 49% of the biomedical PhD workforce ultimately remains in research and teaching careers in academia or the government (3). This is consistent with the report by Golde and Dore, who found that only 36.3% of PhD students in chemistry, 42.9% in molecular biology, and 52.6% in psychology considered a faculty career (4). Indeed, scientists are employed in almost every professional field, including applied research in industry, teaching-intensive careers, public policy, and public or private consulting. Moreover, neuroscience in particular has implications beyond academic medicine and higher education; for example, it intersects with public health, national security, ethics, and law.

Recognizing these facts, the IPN emphasizes the benefits of professional skill development for its students regardless of their anticipated future career direction. This approach is consistent with the increased attention placed on the development of professional skills among scientists. For example, doctoral fellowships and training grants from the National Institutes of Health (NIH; i.e., the National Research Service Award) require training in the ethical conduct of research (5), with career development activities (e.g., to enhance grant-writing or communication) a required component of K-series awards for postdoctoral trainees (6).

## Background: program demographics

The IPN began matriculating students in the fall of 1994, and was thus just shy of a decade old when it joined CID in 2003. More than 50 faculty members, drawn from 11 departments across both the main campus and medical center at GU, are a part of the IPN. As of 2014, the program has 44 enrolled students and has graduated more than 110 alumni. The IPN has matriculated an average of nine students per year over the past decade; 89% of students that matriculated since 2006 entered thesis research. In addition, approximately 12 post-doctoral fellows actively contribute to the program through teaching in courses and in the laboratory. The female to male ratio of the student body is 1.75:1 and under-represented minorities make up 20% of the program. IPN students matriculate with diverse prior experiences, with educational backgrounds ranging from neuroscience, biology, and psychology to engineering, mathematics, and physics. Some students enter immediately after completing a bachelor's degree, whereas others have Master's degrees and/or full-time research experience.

## Background: core coursework

The IPN's interdisciplinary and interdepartmental program reflects the current state of neuroscience as an innately diverse field. Accordingly, the IPN requires that students build a broad base of knowledge. Because students have diverse backgrounds when they matriculate, we offer a yearly summer course (described in *Teaching*) to provide all students with a strong foundation in neuroscience before they initiate core coursework in the fall. Although some may view this diversity as a challenge, we consider it a strength. We have found that a diversity of experience in a given cohort of students allows them to serve as resources for each other during the first-year coursework and provides for diverse approaches to problem solving.

**Fig. 2:**
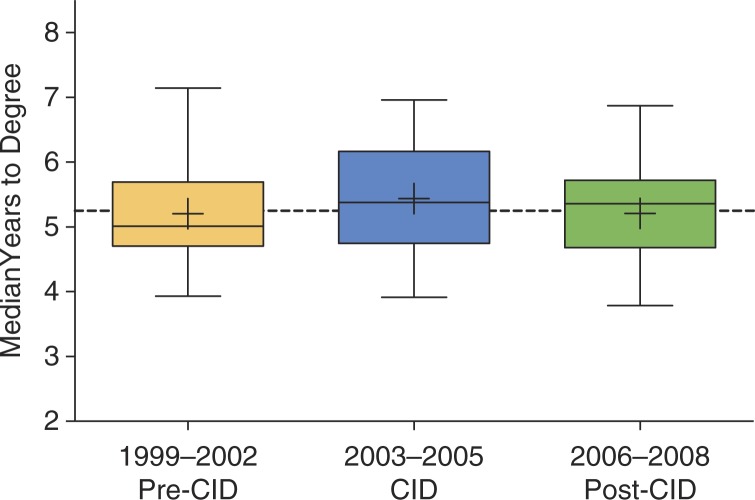
Box and Whisker plots (1st to 99th percentiles) showing time to degree as a function of what epoch of the program students matriculated during (i.e., 1994 to 2002; 2003 to 2005; 2006 to 2008). Dual-degree (MD/PhD) students are not included. The dotted line (5.25 years) shows the overall median.

The first year of graduate school in the IPN consists of three or four semester-long laboratory rotations conducted concurrently with a 1-year sequence of core courses. These core courses are designed to introduce fundamental concepts in neuroscience. As described later, and in Fig. 2, students also are enrolled in courses that develop professional skills. Together, these courses allow IPN students to cultivate their professional identity following a written comprehensive exam taken at the end of the first year of graduate studies.

Students begin thesis research in their second year while concurrently enrolled in elective courses. Aside from at least one course in statistics, no additional courses are specifically required, and the required credits can be met by any number of electives. By the end of the second year, students identify a thesis mentor, and defend a fellowship proposal for their oral comprehensive exam.

Although the principal focus of IPN students during dissertation research is completion of an original research project, students are strongly encouraged to remain engaged with the program outside of the lab to further develop their scientific and professional skills. The success in engaging students in these areas of development is demonstrated by the rate of participation in optional professional development activities (Table 2).

## Professional skills

The seven professional skill domains the IPN strives to develop in its students are: leadership, oral and written communication, teaching, public outreach, ethics, collaboration, and mentorship (Table 1). The definition of a steward includes each of these domains; ‘Taking responsibility for the future of their chosen discipline’ requires leadership and mentorship, ‘dissemination of knowledge’ requires oral and written communication and teaching, ‘sharing … with lay people’ entails public outreach (1). Moreover, the definition of a steward conveys the need for ethical and moral reasoning (7). Finally, in order to become a steward of an interdisciplinary field, collaboration is an essential skill. Training in these skills has been perceived to be lacking in doctoral education in the sciences (8). Others have recommended that graduate education should include structured opportunities to develop broader professional skills (9). In the IPN, the emphasis placed on these skills began with participation in the CID. The specific curricular components developed as a result of CID participation are highlighted in Table 2. These activities are incorporated into required coursework and informal settings, with the intent of maximizing student–faculty involvement.

**Table 1:** Activities by skill domain influenced by CID participation

Professional skill	Activity
Leadership	Student representatives on program committees Student government constitution Retreat planning
Teaching	Creation of Summer course Updating of ‘Drugs Brain Behavior’ course Co-teaching between faculty and students
Public outreach	Financial support for BAW ‘Hot Topics in Health Science’
Ethics	Tea Time Neuroethics Discussion group
Mentorship	Mentorship questionnaire
Oral and written communication	Rubrics for oral comprehensive exams and neurolunch presentations

**Table 2:** Participation rate for individual domains of professional development

Professional skill	Participation rate, %
Leadership	44
Teaching	84
Public outreach	50
Collaboration	57
Ethics	100* (65%)
Mentorship	100*
Oral and written communication	100*

Rate of participation in leadership was defined as the number of students enrolled in 2014 that served in student government at any point during their graduate training. Rate of participation in teaching was calculated as described in (19). Rate of participation in public outreach was defined as the number of students enrolled in 2014 that participated in either: Brain Awareness Week or Hot Topics in Health Sciences. All students are required to take the Skills and Ethics course, giving a 100% participation rate. The number in parenthesis indicates the rate of optional participation, defined as the number of students enrolled in 2014 that participated in either: Tea Time, Neuroethics Discussion Group, the RCR pilot program, or as panelists in the Skills and Ethics. Note that all students also participate in ethics training during their first year. Rate of collaboration was defined as the percent of papers published by IPN graduate students with two or more IPN faculty members as authors between 2006 and 2008. All students participate in the peer mentorship program, Neurolunch presentations, and manuscript writing leading to the 100% participation rate for those domains. Rates were assessed by LEU and PAF in 2014.

* indicates required participation in at least one of the activities described.

## Leadership

IPN faculty members are drawn from a variety of departments and campuses at GU, thus, students serve as a unifying element of the program. This is demonstrated in part by the leadership activities described throughout the manuscript. In fact, students often initiate, plan, and implement program activities. Participation in extra-curricular leadership activities encourages the development of skills in decision-making, teamwork, time management, assertiveness, flexibility, and empathy (10).

Student involvement in program governance grew out of the IPN's participation in CID. There are 14 positions reserved for students on governing committees and in 2014, 44% of the IPN students had served in at least one of these positions. IPN students are involved in program governance beginning in their first year. For example, a first-year student serves as a representative to the IPN Curriculum Committee. Because the core coursework is team-taught by 35 faculty members, IPN students are in a unique position to assess the curriculum as a whole, and offer evidence-based solutions for curricular improvement. This process has involved student-implemented data collection (i.e., formal and informal surveys) and presentations of the data from these surveys to faculty in program leadership.

One example of a student-driven curricular change is the refinement of ‘Critical Readings in Neuroscience’. Originally, this was a literature-based course not directly related to the content of other first-year courses. IPN students recommended linking the critical readings to the topics being concurrently covered in the core neuroscience course. As such, the course now focuses on developing skills to understand and evaluate primary literature with a reading list that mirrors the topics discussed in the neuroscience core course.

IPN students also actively participate in the Executive Committee, which sets policy for the IPN, and is otherwise composed of elected faculty members. The student body elects a voting student member of the Executive Committee. The addition of a voting student member to the Executive Committee required amending the program bylaws, and was approved by a three-fifths majority of faculty. This vote by the faculty underscores the value and trust placed in student input. This responsibility led to students drafting an IPN student government constitution, which details the responsibilities of the elected student officers.

Fostering leadership in IPN students has had effects that extend beyond our program. IPN students are leaders in other areas of student governance and administration. The three presidents of GU's Medical Center Graduate Student Organization (MCGSO) from 2012 to 2014 have been IPN students. Moreover, recognizing a need within the student body for more educational experiences regarding grant writing and review, IPN students designed and implemented a student grant program run through the MCGSO (11).

The active and extensive leadership roles assumed by IPN students form a common thread, tying together the multiple aspects of stewardship discussed in this manuscript. In this way, our students have been a major driving force behind the development, implementation, and refinement of the majority of the initiatives outside of core program requirements.

## Written communication

A steward of the discipline must conduct high-quality science and effectively communicate results to the larger scientific community. Regardless of general writing ability, repeated practice and feedback are necessary to master the specific rhetorical conventions of a field (12); for this reason, and because of the range of specialized forms of communication required for success in science, the development of oral and written communication is emphasized in the IPN.

With respect to written communication, research papers and grant proposals are formal components of coursework, beginning in the first year. Students are introduced to research papers through class discussions that examine the structure of a scientific paper; through this process, students learn effectively communicate written results. Submission of at least one first-author paper is a requirement for graduation. Writing development culminates with the doctoral dissertation.

The majority of faculty in academic medicine report that ‘effective writing of grants and publications’ is their highest career development need (13). Because successful grant proposals are an enabling component of a productive research career (14–16), development of grant writing skills begins early in doctoral training in the IPN. During their first semester, students take ‘Survey of Neuroscience’; this course focuses on critical reading and evaluation of faculty grant proposals. This simultaneously exposes students to research opportunities within the program and to the style and nuances of grant writing; the grade in the course is based on a grant proposal written by the student.

Students write a second proposal in the spring, in the form of an NIH fellowship application. The proposal is reviewed in a student-driven study section modeled after NIH's grant review process. Participating as a grant reviewer allows students to reflect on the decisions they make in their own grant writing. Finally, the second-year comprehensive exams take the format of a grant proposal that is orally defended to a panel of faculty.

## Oral communication

Development of oral communication skills is also a recurring theme throughout students’ time in the program. Students present in journal clubs and participate in discussions with seminar speakers throughout their time in the program. Moreover, in a required skills and ethics course (taken during their second semester), poster presentation skills are discussed and student presentations are critiqued by a public speaking coach.

Students are also required to present their rotation research projects to the program after each rotation during their first year, and annually during thesis research. Students are given formal feedback on their presentations (including their oral comprehensive examination) through a rubric completed by members of their thesis committee. Rubrics were implemented to increase the frequency of feedback, and because the use of rubrics has been associated with higher achievement and deeper learning by students (17). IPN students also have the opportunity to participate in a medical-center-wide ‘Student Research Day’ poster session. These in-house activities supplement expected presentations of posters or oral presentations at conferences. The final oral component, as is typical for PhD programs, is the thesis defense.

The vital role of communication extends beyond simply conveying newly generated knowledge, and includes conveying existing knowledge of the field in the classroom and in outreach settings, two facets of professional development that we discuss below.

## Teaching

A key component of stewardship is integrating knowledge into the current framework of the discipline, and conserving and transforming that knowledge by conveying it to colleagues, students, and laypeople. This is at the heart of effective teaching. Although teaching is not mandatory for students in the IPN, as of 2014, 84% of the thesis students participated in teaching. Time commitments ranged from several hours a semester, to course directorship (50 or more hours per semester). Once a semester, the IPN sponsors an evening discussion session led by a rotating group of faculty members to discuss pedagogical issues, identify areas of concern, and to provide oversight and support to student teaching.

To assess attitudes and opinions regarding student teaching, we conducted a survey of IPN training faculty who had mentored at least one thesis student between 2003 and 2013. We obtained responses from 35 faculty members (response rate: 87.5%). More than 90% of respondents agreed that PhD students should have opportunities to gain teaching experience as part of their doctoral training, and more than 90% agreed that PhD thesis students in their lab were welcome to spend time teaching, assuming it does not interfere with the progression of their research. Recognizing the importance of teaching as a component of scholarship, 88% of respondents felt that teaching in an area related to thesis research is a good way for students to enhance their overall scholarship, and 70% felt that having a teaching portfolio developed as a graduate student would provide a competitive advantage when seeking jobs. These attitudes are reinforced by the finding that teaching experience is associated with improved research skills (18). Because of student desire and a highly supportive faculty, teaching – and learning to teach – has become integral part of the IPN.

The teaching opportunity that has the most formal structure is ‘Drugs, the Brain, and Behavior’, an elective course for undergraduate and MS students; we have previously described this course in detail (19). Although this course was initially developed prior to CID, as a result of CID it was extensively reformed (19). It is team-taught by PhD candidates in the IPN and directed by two to four students in their third or fourth year in the program. CID-driven revisions of the course include: oversight provided by a steering committee of IPN faculty and an emphasis on development of pedagogical skills for PhD candidates who are teaching. This involves use of classroom technology, formal and informal evaluation by course directors, feedback provided by faculty steering committee members on video-recorded lecturers, and a one-credit teaching practicum. In the teaching practicum, which is a prerequisite to be eligible to direct the course, students observe half of the lectures each semester and write responses to each lecture focusing on pedagogical strategies.

IPN students also take on leadership roles in several courses that are part of the core curriculum; these opportunities began during CID participation and have continued to grow since that time. For example, senior students co-teach a summer course to prepare incoming students for the first-year curriculum and promote early interaction between incoming students, senior graduate students, and faculty. The experience concludes with a series of hands on ‘boot camp’ sessions in which students conduct experiments to gain an understanding of methods and explore neuroanatomy in human and animal specimens. More than 40 students have taught in this course over the past 4 years.

Because the core course in neuroscience is team-taught, integrating information across faculty and modules can pose a challenge for students encountering the information for the first time. To address this concern, a student-taught, mandatory (one credit) recitation session meets weekly and includes review sessions prior to exams. It focuses on reviewing information taught in the core course and setting it in the broader framework of neuroscience. Three students led this course between 2011 and 2014.

A key theme of all of these opportunities is the hands-on involvement of the IPN graduate students in teaching, with an emphasis on responsibility, pedagogy, and complementary teaching styles. This training, which remains rare in PhD programs (4, 20, 21), is aimed at the early development of teaching as a core component of professional identity, with the intent that it should benefit students regardless of eventual career path.

## Public outreach

As a steward of the discipline, a scientist must convey information not only to students and colleagues, but also to the general public. As the Royal Society recognized in 1985, ‘Almost all public policy issues have scientific or technological implications. Everybody, therefore, needs some understanding of science, its accomplishments, and its limitations’ (22). Public access to scientific information is growing – for example, in 2008, NIH established PubMed Central to increase public access to research supported by public funds. However, the extent to which the public makes use of digital archives of full-text, peer-reviewed journal articles to increase their understanding of science remains unclear, with data suggesting that users are more likely to get their information from secondary sources such as Wikipedia or MedlinePlus (23). Because non-experts believe scientific findings to be more satisfying and reliable when the explanations include a neural basis for the reported results (24), neuroscience may be particularly affected by the public reliance on non-authoritative sources. This underscores the importance of public outreach to dispel misconceptions.

Interestingly, participation in public outreach has also been shown to be positively correlated with higher research productivity (25). Recognizing this, IPN students are highly encouraged to become involved in opportunities involving informal teaching and public outreach. Fifty percent of IPN students participate in public outreach activities.

Some examples of student-driven public outreach follow. IPN students and faculty regularly participate in Brain Awareness Week (BAW), a global campaign to increase public awareness of the progress and benefits of brain research. The IPN has participated in BAW since its founding by The Dana Alliance for Brain Initiatives in 1996. However, after participating in CID, there was an increased emphasis and value placed on BAW participation. This is reflected in monetary support for BAW activities by the program that began after CID participation.

In 2012, the IPN hosted 100 seventh and eighth graders and in 2014, the IPN hosted 115 seventh graders for lab tours and demonstrations about the brain and neuroscience. These activities illustrated various concepts in anatomy, sensation, perception, and attention. When surveyed after the 2012 session, the graduate students who participated in BAW thought it was successful. All graduate student participants indicated that they would be willing to participate the following year, and all rated the day as either ‘successful’ or ‘highly successful’ on a Likert-like scale ranging from completely unsuccessful to highly successful. Moreover, the perception of graduate students that participated as group leaders (and thus visited multiple stations with their students) was that the activities were age-appropriate (59% of responses), informative (63% of responses), and entertaining (68% of responses).

A second outreach activity is ‘Hot Topics in Health Science’; a public seminar series developed by IPN graduate students in conjunction with the District of Columbia Public Libraries. During these regularly scheduled library programs, a graduate student, post-doctoral fellow, or faculty member presents a current issue in health science to a lay audience. Seminar topics have ranged from ‘Food for Thought: How Your Diet Can Help You Get the Most Out of Your Brain’ to ‘Pandemic Flu: Birds, Pigs, and Planes’. To ensure that the series’ goals are being met, each seminar is video- and audio-recorded, both for review by the speaker as well as for dissemination via podcast. Underscoring the ability of presenters to effectively translate these topics for a lay audience, surveys showed that more than 90% of the audience felt there was an appropriate level of detail and more than 80% felt the seminars were ‘useful’ or ‘very useful’.

In addition to conveying information to lay, student, and peer audiences, stewardship requires the consideration of the impact of knowledge, and more broadly, ongoing research, on society at large. This is one of the many areas of study of the burgeoning field of neuroethics.

## Ethics

Ethical issues in science range from the responsible conduct of research (RCR) to how research has an impact on society. Through a combination of coursework, discussion groups, and publication opportunities, IPN students are able to engage with faculty, peers, and outside community members to explore a variety of ethical issues. All IPN students are required to participate in ethics training.

In addition to the practical skills emphasized in the ‘Skills and Ethics’ course (as described previously in the *Oral and Written Communication* section); this course also engages students in discussions regarding the RCR, scientific ethics, and ethical dilemmas inherent to the scientific community. This is particularly important given increasing reports of compromised ethics in conducting research and reporting of results (26, 27). The course follows principles and practices that have been outlined in detail by Fischer and Zigmond (28–30), including integration of RCR and professional skills training in the same course, the use of ethics cases, small-group discussions, faculty-student interaction, and a less formal setting to encourage open discussion.

Topics range from data management to policies governing grant and manuscript review to the ethics of authorship (31). Because authorship practices may be inconsistent from lab to lab, discussion of the stated and agreed-upon policy of the journals (32) is particularly important. By providing students a forum to discuss the various challenges associated with authorship early in their career, students are able to exercise these lessons while they plan, write, and submit their own publications in graduate school.

In addition to the formal discussions of ethics in this course, topics related to the ethical conduct of science are woven throughout the first-year curriculum, ranging from the discussion of appropriate controls in journal clubs and seminars to real-world ethical dilemmas encountered by faculty during presentations in the Survey course. There are also several optional extracurricular opportunities through which students explore ethics; 65% of students have participated in non-mandatory ethics opportunities.

Some students have also participated in an optional RCR pilot program following a mastery rubric developed at GU (33). In this program, students are presented with issues raised continually in biomedical research – including issues of informed consent, data sharing, and animal use and care. Assignments facilitate the development of critical thinking regarding these issues.

During and after participation in CID, IPN students designed two forums to discuss ethical issues: the Neuroethics and Tea Time discussion groups. In the Neuroethics discussion group, IPN students explore neuroethics and neurophilosophy through presentations that foster discussion among fellow IPN students and faculty. Speakers have led sessions on the ethical use of animals for research, applying neuroscience research to the battlefield, and the conception and philosophy of ‘the self’. Additionally, students have the opportunity to develop these ideas more fully and assemble these arguments into a paper for publication.

The Tea Time forum provides students and faculty a place to discuss current events in neuroscience in an informal environment. Discussions have included: effective communication between mentor and mentee, balancing family and career, and ongoing issues in the news and public domain. Tea Time includes discussions on the perspective of audiences such as: researchers from other scientific disciplines, members of the media, the legal community, government leaders, and the public.

These forums are examples of the collaborative environment fostered in the IPN: students and faculty work together to shape presentations and discussions of mutual interest. This collaborative spirit extends into the laboratory and even beyond the Georgetown campus, as described below.

## Collaboration

Science is an inherently collaborative enterprise, building on the research and ideas of the past to create new knowledge. As the field of neuroscience continues to expand, encompassing aspects of physics to psychology, an individual scientist is less able to develop expertise in the discipline as a whole; thus, collaboration plays an increasingly important role in ameliorating the pitfalls of overspecialization and increasing amounts of data (34). High levels of collaboration are also associated with high research productivity, as defined by the number of publications (35). The IPN attempts to foster the spirit of collaboration early in the program, encouraging the exchange of ideas, knowledge, and skills within GU and with outside collaborators.

As detailed above, students are introduced to collaboration by actively engaging with outside speakers during seminars and journal clubs. Students are encouraged to take advantage of these opportunities to develop relationships with scientists at other institutions. Many students use these events to identify an external member of their thesis committee, begin research collaborations, or secure post-doctoral positions.

Students are also encouraged to do a fourth research rotation outside of GU. Students have used this opportunity to work in labs from Australia to Japan to the Netherlands. Students are welcome to establish their own contacts or take advantage of already-established partnerships, such as the International Research Training Group, which exists between several neuroscience labs in Munich, Germany and GU.

One exceptional opportunity for IPN students is the Partnerships for International Research and Education program funded by NSF, which seeks to establish innovative models for international collaborative research and education. This program allows students to conduct their thesis research collaboratively with a laboratory at GU and a laboratory in Europe. Several students have used the program to forge international collaborations with labs in Germany and Finland, and have also used these collaborations to secure future post-doctoral positions. All of these programs and opportunities not only build relationships with other labs and institutions, but also strengthen the scientific capabilities at GU as IPN students return with new techniques, skills, and knowledge to disseminate.

Collaboration within GU is also emphasized. Since 2005, the IPN has held a yearly 2-day retreat for faculty and students to discuss science informally and socialize. The absence of community has been identified as a factor contributing to student attrition in doctoral programs (36); events like the retreat help foster a sense of community and provide a support structure for IPN students. The retreat is also used as an opportunity to evaluate the past year and discuss the future of the program. The retreat continues to play a major role in maintaining the close-knit nature of the IPN community and creating an academic environment conducive to collaboration. Importantly, the retreat was developed as a direct result of CID participation.

As part of this effort, students are encouraged to form co-mentorships for their thesis research. During each epoch of the IPN, formal co-mentorship rates were constant at 23–24% of students. Informal collaboration, as measured by the percent of papers published by IPN graduate students with two or more IPN faculty members as authors during a given epoch, was 33% prior to CID participation, 32% during CID participation, and 57% in the post-CID period. Students often act as the catalyst for new and productive partnerships between faculty, spurring new avenues of research or new grant proposals. For papers that had two or more faculty authors, we identified the subset in which the faculty authors had not previously published together. This subset was used to calculate the number of new collaborations per student. This rate was 0.29 prior to the CID, 0.28 during the CID, and 0.5 after the CID.

## Mentorship

In the IPN, a variety of relationships are cultivated to facilitate the transformation from student to steward. Students serve as both mentees and mentors during their tenure in the IPN. Peer mentorship has been associated with increased involvement in graduate program activities, including social and leadership components (37). Moreover, structured mentorship programs between trainees and faculty has been associated with improved research productivity (38, 39). All IPN students participate in peer mentorship during their time in the program. Mentorship relationships are developed with faculty members, fellow students, and members of their own lab. An integral part of IPN students’ training is to form these connections ‘at the bench’, in the classroom, and beyond.

Months before coming to GU to begin their doctoral work, incoming students are contacted by a peer mentor who offers advice on moving to Washington, DC, and is available to answer any questions the incoming student may have. Peer support is beneficial for students, especially early in the graduate career, and is higher in departments and programs with higher student completion rates (40). At GU, peer mentors are a built-in support system these students can count on from day one to offer insights into the ins and outs of graduate school. Once they arrive at GU, first-year students also often form informal mentor-mentee relationships with more advanced students. These relationships are facilitated by interactions with graduate student instructors, monthly student meetings, and the annual IPN retreat.

Perhaps the most important type of mentoring that occurs during graduate training is between a student and his or her thesis advisor(s) (41). Poor mentorship has been cited as a prominent cause of attrition in doctoral students (36). Moreover, dissatisfaction with thesis advisors is also associated with decreased engagement in professional activities (36). Because of the importance of this relationship, we have developed and implemented a mentor questionnaire to facilitate open communication between students and their thesis advisor(s) (Supplementary file 1). This document highlights areas that should be discussed, including expectations of the mentor and mentee in terms of: time commitment, publications, student responsibility, independence, and benchmarks of productivity. Its use is encouraged, particularly during the stage when students and faculty are evaluating each other as a potential fit for this role. This questionnaire was developed through a series of discussions at the annual retreats, and is an indirect result of CID participation. Feedback from both the faculty and students has been extremely positive, indicating that it aids in broaching topics that can often be awkward to approach, such as the availability of funding for the student.

The benefits of mentorship for graduate and post-doctoral trainees has been previously assessed in a small sample (*n=*8) of molecular biologists; although difficult to generalize with such small samples, the authors found that these junior mentors reported gains in research productivity and quality, interpersonal skills (including mentoring skills and communication skills), socio-emotional benefits, and increased understanding of the responsibilities of a faculty member (42). The general literature on mentorship also suggests that it provides significant personal and professional benefits for both mentees and mentors (43). Recognizing these benefits, as students enter their advanced years of graduate study, they are encouraged to become a mentor. They often take on a peer mentee, as described above, or provide guidance to undergraduate student(s) or research assistant(s) in their laboratory. As stewards of the discipline, IPN students take the knowledge and experiences they acquire as a mentee and put those into practice as they begin mentoring the next generation of trainees in the scientific community.

## Faculty perspective

Although the program developments discussed above have focused on nurturing skills and professional identity in our students, we believe that the entire deliberative process has been beneficial to IPN faculty. For example, during the development of a mentorship survey, faculty discovered that colleagues did not necessarily share many of their own assumptions about mentor-mentee responsibilities. Diversity in faculty perceptions regarding mentor-mentee responsibilities have been reported by others (44), underscoring the need for clear communication about expectations. In addition, the efforts to foster research collaborations led students to directly collaborate with each other across labs and seek out co-mentors. In this way, highly productive new research directions leading to new grants and publications have been established between faculty members who had not previously worked together. This is consistent with the view outlined in a recent NIGMS report that, ‘many investigators believe that research training and laboratory productivity are synergistic’ (45).

An especially valuable type of student–faculty collaboration developed as an outgrowth of the CID: teaching partnerships in which faculty and students worked together to design lectures, assignments, exams, and even full courses, with several student–faculty pairs teaching classes for the program. In a typical year, approximately 25 students and 10 faculty members participate in team-teaching. This shared activity improved the pedagogical skills of both students and faculty, and introduced the faculty to novel teaching technologies and strategies such as the use of iClickers and SmartBoards. In addition, it allowed faculty to discover how effective the students are as teachers, raising appreciation for the value of having students engage deeply in classroom teaching. These attitudes are reflected in the faculty survey on student-teaching (see *Teaching*).

Informal discussions among faculty have underscored an intangible benefit of CID participation: allowing faculty to gain a deeper insight into the capabilities of the students as creative and responsible contributors to the shaping and administration of the graduate program. This realization is reflected in the fact that faculty have welcomed students as colleagues in the design and revision of policies and activities. This has fostered an atmosphere of mutual trust, with frank and open communication. This positive spiral enables a powerful sense of joint ownership of the program by students and faculty, a feature that has been maintained and nurtured to the present time.

## Metrics

The primary metric for the seven professional skills we have described is participation. Participation rates in each skill domain are listed in Table 2. The majority of the activities listed are optional, so participation reflects the value placed on skill development by the IPN students and faculty. Because the first class to matriculate after CID participation has graduated as of 2014, we do not have a sufficient sample to compare long-term outcomes of these initiatives. However, we have analyzed standard metrics of graduate program success, such as time to degree, number of publications, and post-graduate career path.

We divided all graduates of the program into three bins based on time period in which they matriculated (i.e., before participation in the CID, during the CID, and after the CID). Because many of the program reforms we describe were implemented during the participation in CID (2003–2005), the effects of these programs should be detectable in students that entered after its completion. For the post-CID period, we only included students who matriculated between 2006 and 2008 because students entering in 2009 onward have not graduated as of 2014. As we have described, the IPN curriculum underwent major revisions during CID participation; thus, we have collapsed all pre-CID data for analysis.

As shown in Fig. 2, time to degree has been stable for students that matriculated across the three major epochs of our program, the pre-CID years, the years of active CID participation, and the years after CID participation (Kruskal–Wallis Test: *H*=1.2, *P*=0.57). The median years to degree does not differ significantly from those reported in the most recent Society for Neuroscience Committee on Neuroscience Departments and Programs (CNDP) survey (2) and those reported in the National Academy of Sciences assessment of the research doctorate (46) (Wilcoxon Signed Rank Test: Ps>0.30). Thus, the professional development activities we have described herein do not present an obstacle to timely completion of the PhD.

**Fig. 3:**
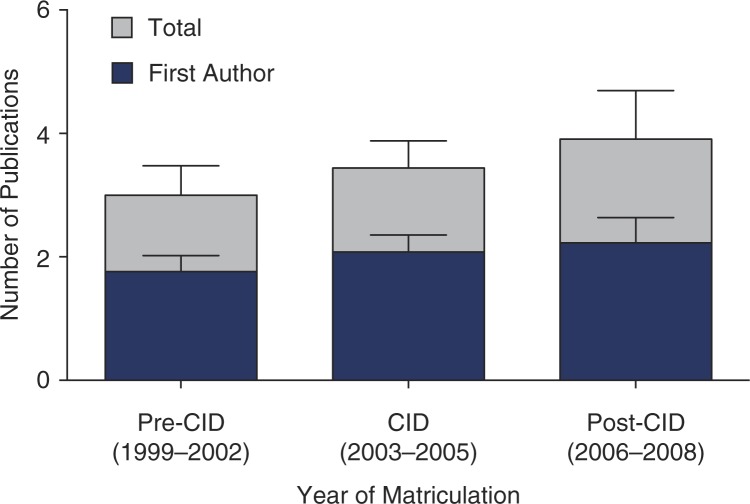
Mean (and standard error) of the number of publications (total publications in grey, 1st author publications in blue) produced by IPN students as a function of period of matriculation.

As shown in Fig. 3, students who matriculated during the pre-CID period published a mean of 3.0 manuscripts (1.8 first author), during the CID period, students published a mean of 3.4 manuscripts (2.1 first author), and during the post-CID period, students published a mean of 3.9 manuscripts (2.2 first author). Interestingly, the number of publications did not differ statistically as a function of period of matriculation (*F*_2,69_=0.62, *p*=0.5 for total publications; *F*_2,69_=0.57, *p*=0.6 for first author publications).

**Fig. 4:**
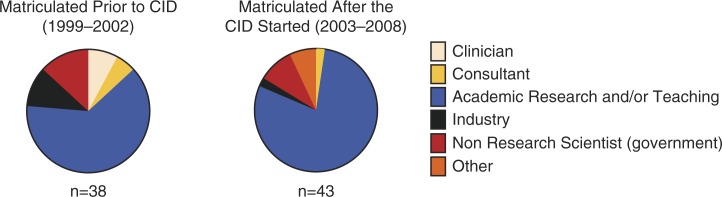
Career path after the completion of degree as a function of what epoch of the program students matriculated during (i.e., 1999 to 2002; 2003 to 2008). 1999 was selected as the cutoff for this to allow an assessment of an approximately equal number of students. This is also the period during which the program initially received funding through the NIH Jointly-Sponsored Predoctoral Training Grant program.

Figure 4 shows the relative distributions of current post-PhD careers of IPN graduates as a function of time of matriculation (pre- and post-CID). In order to compare an equivalent number of graduates, we collapsed the data from the during-CID and post-CID periods of matriculation. It is worthy of note that the majority (72%, collapsed across time-periods) of our students go on to post-doctoral or other academic research positions immediately after the PhD, a number consistent with that reported by the 2011 CNDP survey.

In line with reports from others (47), the above data indicate that participation in professional development activities does not detract from traditional metrics of graduate student success.

## Conclusions

We have highlighted here seven professional skills which the IPN strives to develop in its students: leadership, oral and written communication, teaching, public outreach, ethics, collaboration, and mentorship. We have described these foci, which emerged largely from the IPN's participation in the CID, as well as their benefits to the students and faculty. Through a variety of formalized activities, as well as by fostering a community that values these traits, we encourage IPN students to become stewards of the discipline, not only by generating new knowledge, but also by integrating that knowledge into the current framework of the discipline and conserving and transforming the knowledge by conveying it to colleagues, students, and laypeople.
